# Calcitonin receptor is required for T-antigen-induced prostate carcinogenesis

**DOI:** 10.18632/oncotarget.27495

**Published:** 2020-03-03

**Authors:** Ajay Kale, Afaf Aldahish, Girish Shah

**Affiliations:** ^1^Pharmacology, University of Louisiana College of Pharmacy, Monroe, LA 71201, USA

**Keywords:** calcitonin receptor, T-antigen, tumor growth, PTEN, prostate cancer

## Abstract

Expression of calcitonin (CT) and its receptor (CTR) is frequently elevated in prostate cancer (PC) and activation of CT–CTR axis in non- invasive PC cells induces an invasive phenotype. However, the role of CT-CTR axis in prostate carcinogenesis has not been investigated. We employed a transgenic mouse prostate cancer model that uses long probasin promoter to target the expression of T-antigen in the prostate gland (LPB-Tag) along with CTR knock-out mice (CTRKO) to address this question. We cross-bred LPB-Tag mice with CTRKO to obtain four groups of mice. Prostates of these mice were obtained at the age of 90 days, fixed, paraffin-embedded, and used either for the extraction of RNA or for immunofluorescence. Prostate RNAs from different groups were reverse transcribed and used either for transcription profiling or for qRT-PCR. As expected, prostates of mice with LPB-Tag genotype displayed well-grown tumors with histologic features such as loss of normal morphology and nuclear atypia. WT as well as CTRKO mice displayed normal prostate morphology. Interestingly, LPB-Tag-CTRKO prostates also displayed relatively normal morphology which was indistinguishable from the WT. Microarray analysis as well as qRT-PCR suggested that CTRKO genotype reversed T-antigen-induced silencing of RB and PTEN gene expression as well as T-antigen-induced expression of several enzymes associated with lipid metabolism/ cholesterol biosynthesis, several cancer-related and androgen-regulated genes. The results for the first time identify mechanisms associated CTR-induced prostate carcinogenesis, and raise an exciting possibility of using a potent CT antagonist to attenuate progression of prostate cancer.

## INTRODUCTION

The Calcitonin Receptor (CTR) is a G protein-coupled cell surface receptor that is expressed in osteoclasts, renal, neural cells, and peripheral organs like mammary and prostate glands, and signals through the activation of adenylate cyclase and phospholipase C [1–4]. Calcitonin (CT), a 32-amino acid peptide, is a natural ligand for CTR and the most potent inhibitor of osteoclastic bone resorption [1]. CT is secreted primarily by thyroid C cells in response to elevated serum calcium levels but is also secreted by neural and peripheral tissues including the prostate gland [5]. The main recognized actions of CT are calcium homeostasis, inhibition of bone resorption, reduction of calcium tubular reabsorption, and the regulation of 1, 25(OH)_2_D_3_ production in the kidney [2, 6]. However, the expression of CT/CTR in multiple organs and their actions in development, cell growth, and differentiation suggest that CT/CTR may have a more diverse role in human development and diseases [6–8].

Expression of CT and CTR transcripts is elevated in malignant prostates, and correlates positively with Gleason grade of prostate cancer (PC) [3]. Moreover, activation of the CT-CTR autocrine axis stimulates processes associated with tumor growth and metastasis such as invasion, angiogenesis, chemoresistance, and metastasis [4, 9–12]. CTR also destabilizes tight and adherens junctions, induces epithelial to mesenchymal transition (EMT), and activates non-G protein-coupled signaling pathways such as PI-3-kinase (PI3K)-Akt-survivin and WNT/β-catenin [11, 13]. Our recent studies suggest that the cytoplasmic (C) tail of CTR associates with tight junction (TJ) protein Zonula Occludens-1 (ZO-1) via the interaction between the type 1 PDZ-binding motif in the carboxy-terminus of CTR and PDZ3 domain of ZO-1 [14]. This interaction is critical for the actions of CTR on tight junction destabilization as well as for the formation of distant metastases of orthotopically implanted prostate cancer cells in nude mice. This suggests that the CT-CTR autocrine axis plays an important role in prostate cancer progression and metastasis.

The primary goal of the present study was to examine the role of CTR in prostate carcinogenesis. For this, we employed CTR-knock-out mice (CTRKO) and an established transgenic mouse prostate cancer model that uses long probasin promoter to target the expression of T-antigen in the prostate gland (LPB-Tag). We used the 12T-7f subline of this model, which has been shown to develop prostate tumors much faster [15].

## RESULTS

### Time course of prostate tumor formation in LPB-Tag mice

In our preliminary study, we evaluated the development of prostate tumors at different ages between post-natal 60–90 days. 3 out of 15 mice developed prostate tumors at the age of 60 days; 9 out of 15 developed prostate tumors when sacrificed at day 75; and 15 out of 15 displayed prostate tumors when sacrificed at day 90. Therefore, all mice in the present study were sacrificed at the age of 90 days.

### Phenotypical changes in CTRKO mice

The information provided by the vendors as well as our observations suggested that male CTRKO mice did not display any remarkable phenotypic or behavioral changes during experimental period when compared with their age- and gender-matched wild-type mice. Moreover, CTRKO male and female mice were fertile and their progeny were viable until weaning.

### Current study

A total of 32 animals were used and were divided into following four groups: 1) WT controls (CTRKO-, LPBTag-, *n* = 10); 2) LPB-Tag (LPB-Tag+, CTRKO-, *n* = 10); 3) CTRKO (LPB-Tag-, CTRKO+, *n* = 6); and 4) LPB-Tag-CTRKO (LPB-Tag+, CTRKO+, *n* = 6). At the necropsy, their prostates were harvested, fixed, paraffin-embedded, and fixed. Tumors were either used for RNA extractions or for immunofluorescence studies.

### Changes in body weight and prostate weight

**Figure 1:**
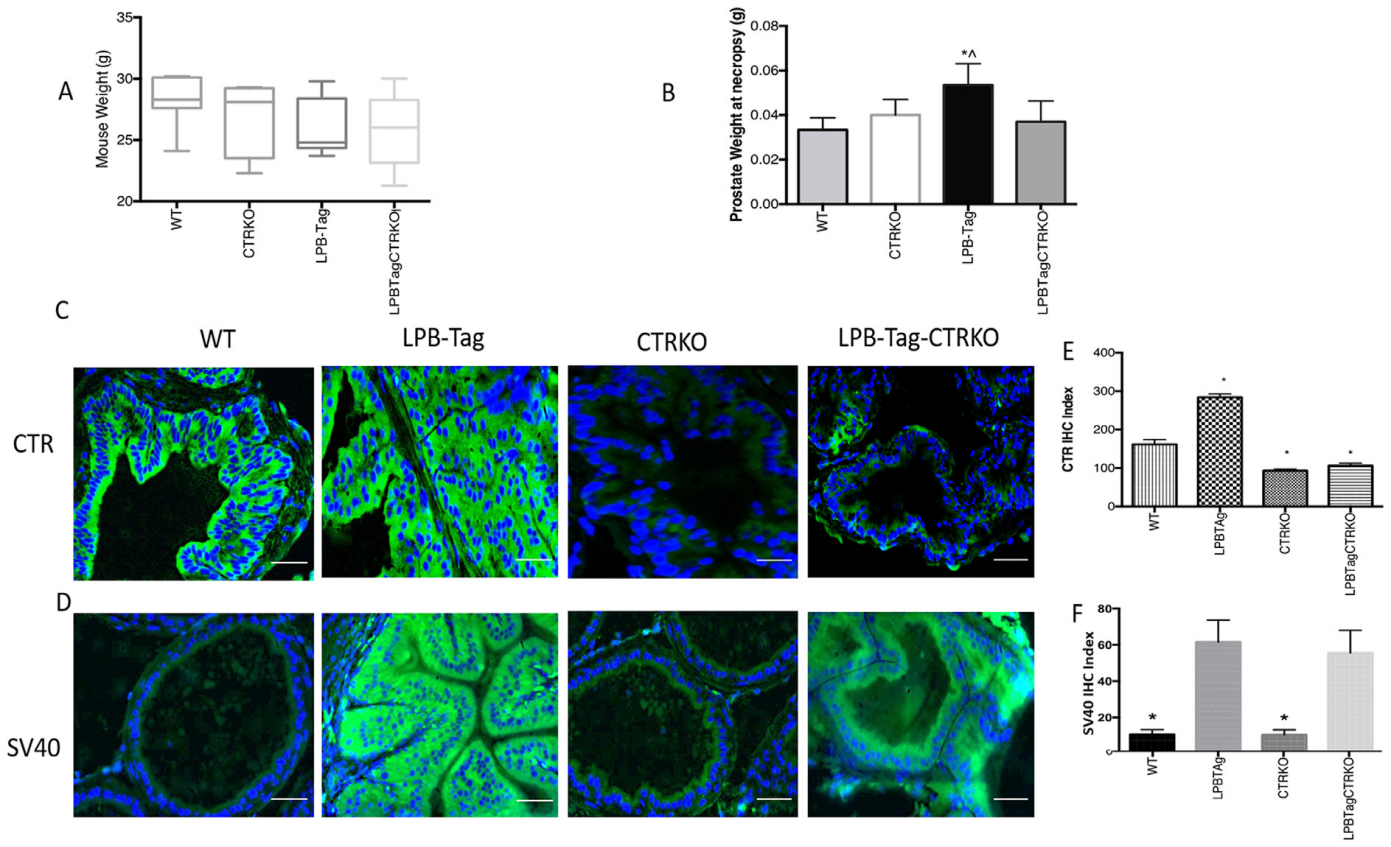
Changes in body weight, prostate weight, and CTR expression. (**A**) Figure represents age matched body weight of WT, CTRKO, LPB-Tag, and LPB-Tag-CTRKO male mice. (**B**) Figure represents weight of prostate gland at the age 90 days for WT, CTRKO, LPB-Tag, and LPB-Tag-CTRKO mice at necropsy. ^*^ represents significantly different than WT, ^^^ represents significantly different than LPB-Tag-CTRKO; *p* < 0.05. (**C**) Representative photomicrographs of immunofluorescence for CTR in the prostate tissues of WT, CTRKO, LPB-Tag, and LPB-Tag-CTRKO mice. Green staining represents CTR activity while blue staining represents the DAPI at 40× magnification; Scale bar 100 µm. (**D**) Representative photomicrographs of immunofluorescence for T-antigen (SV40) in the prostate tissue of WT, CTRKO, LPB-Tag, and LPB-Tag-CTRKO mice. Green staining represents CTR activity while blue staining represents the DAPI at 40× magnification; Scale bar 100 µm. (**E**) Figure represents mean IHC staining index for the CTR immunofluorescence observed in the prostate tissues of WT, CTRKO, LPB-Tag, and LPB-Tag-CTRKO mice; ^*^ represents significantly different than WT; *p* < 0.05. (**F**) Figure represents mean IHC staining index for the SV40 immunofluorescence observed in the prostate tissues of WT, CTRKO, LPB-Tag, and LPB-Tag-CTRKO mice; ^*^ represents significantly different than WT; *p* < 0.05.

Although LPB-Tag and LPB-Tag-CTRKO male mice displayed slightly lesser body weights as compared to their age-matched WT mice, the differences were not significant (Figure 1A). Moreover, their prostate gland weights at the age of 90 days were comparable to those of their wild type littermates (Figure 1B). In contrast, the LPB-Tag animals displayed lower body weights but much larger prostates at the necropsy (Figure 1A and 1B). The prostate weights of both, CTRKO and CTRKO-LPB-Tag mice were closer to those of wild type mice (Figure 1B).

### CTRKO mice lack prostate CTR expression

The absence of CTR in CTRKO mice was further confirmed by immunofluorescence (Figure 1C). The results show that CTR immunoreactivity was abundant in the prostates of WT mice and increased remarkably in LPB-Tag mice. However, CTR expression in the prostates of CTRKO genotype was abolished whereas that of CTRKO-LPB-Tag genotype was greatly diminished. The bar graph of Figure 1E presents pooled quantitative results of CTR immunofluorescence in these samples.

### Presence of CTRKO transgene does not alter T-antigen expression

Intense expression of SV40 (T antigen Tag) was observed in all of the LPB-Tag mice (Figure 1D). Similarly, the expression was also abundant in the epithelia of the prostates of LPB-Tag-CTRKO mice. As expected, the staining was absent in the prostates of CTRKO as well as WT mice. The bar graph of Figure 1F presents pooled quantitative results of Tag immunofluorescence in these samples, and it is consistent with the profiles of representative micrographs of each group.

### CTRKO genotype attenuates T-antigen-mediated tumor formation in LPB-Tag mice

**Figure 2:**
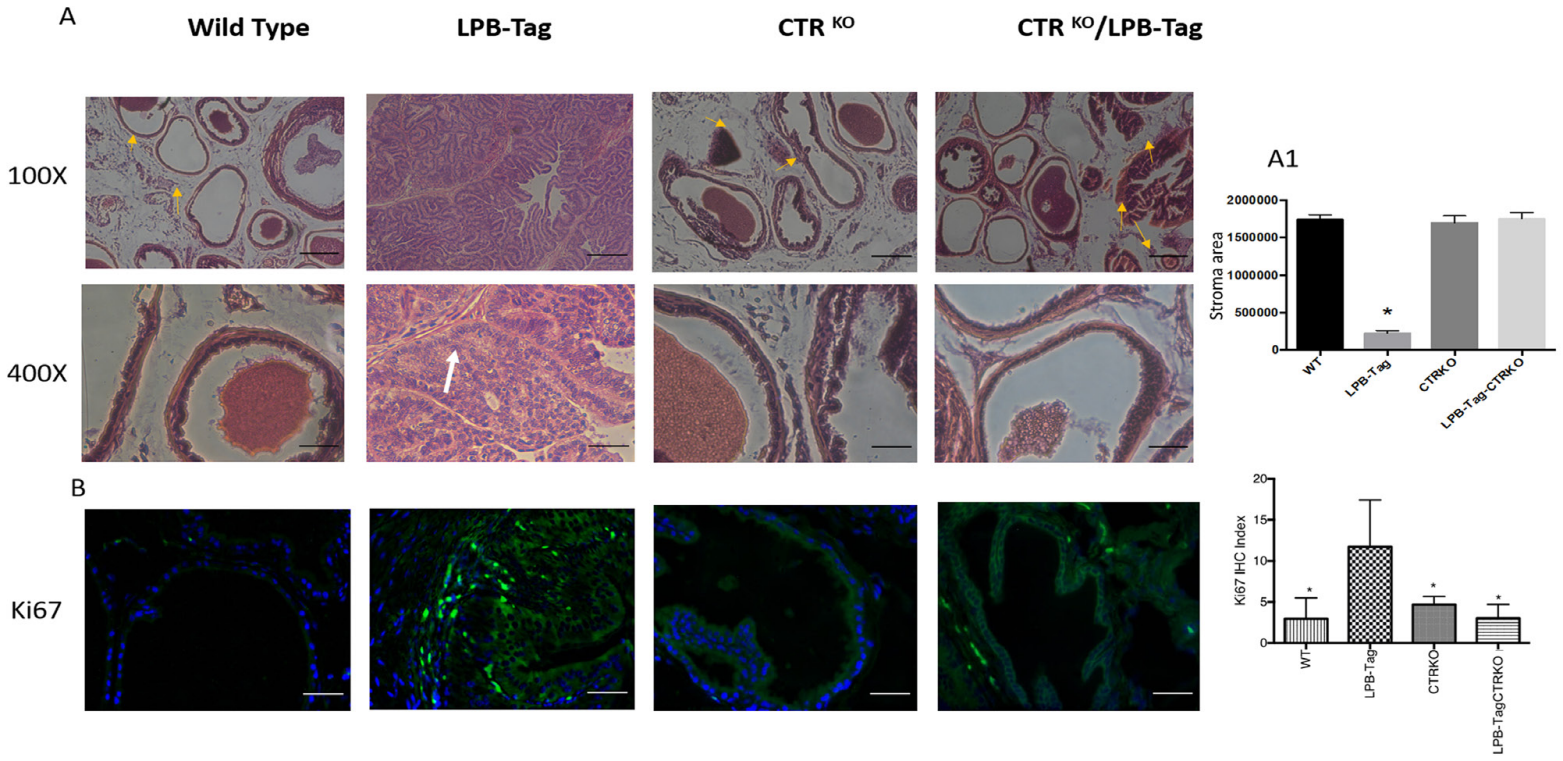
H & E staining of prostate tissue and Ki67 immunoreactivity. (**A**) Representative photomicrographs of H&E stained prostate tissues of WT, CTRKO, LPB-Tag, and LPB-Tag-CTRKO mice; Magnification 100× and 400×; Fibromuscular stroma surrounded by individual gland indicated by arrows; Scale bar 100 µm. (**A1**) Quantitative representation for area of stroma in the prostate tissues of WT, CTRKO, LPB-Tag, and LPB-Tag-CTRKO mice. ^*^ represents significantly different than WT, CTRKO, and LPB-TagCTRKO; *p* < 0.05. (**B**) Representative photomicrographs of immunofluorescence for Ki67 in the prostate tissue of WT, CTRKO, LPB-Tag, and LPB-Tag-CTRKO mice. Green staining represents CTR activity while blue staining represents the DAPI at 40× magnification. The graph represents the mean IHC index for the Ki67 immunofluorescence observed in the prostate tissues. ^*^ represents significantly different than LPB-Tag; *p* < 0.05; Scale bar 100 µm.

H&E histology of WT mouse prostate presented a typical adult prostate morphology, a thin rim of fibromuscular stroma surrounded by individual glands (*arrowheads*), with loose connective tissue extending between individual gland profiles (Figure 2A). In contrast, prostate sections of LPB-Tag mice displayed a total loss of normal morphology with epithelial stratification and nuclear atypia. The prostates of CTRKO displayed glands embedded in fibromuscular stroma. Stroma seemed more extensive than those in WT prostates. However, quantitative estimate of stroma in these sections did not reveal significant differences in the stromal compartments of WT and CTRKO mice (Figure 2A1). Interestingly, the prostates of LPB-Tag-CTRKO mice showed dramatic reversal of the histopathologic modification seen in LPB-Tag prostates, many approaching near normal appearance of the prostate of the CTRKO mice and none of them presented detectable invasive adenocarcinoma features, but also displayed extensive presence of fibromuscular stroma (Figure 2A). These findings indicate that CTR deficiency abolished T-antigen-induced tumor formation in the prostates of LPB-Tag mice.

Hyperplastic and dysplastic conditions in prostate epithelia of LPB-Tag genotype were further confirmed with Ki67 staining. As depicted in Figure 2B, only LPB-Tag mice displayed significant number of Ki-67-positive cells, the staining was nuclear and these cells were predominantly localized in the epithelium. Interestingly, the prostates of all other groups displayed none or minimal nuclear Ki67 staining, further confirming the absence of tumors in these groups, including LPB-Tag-CTRKO mice.

### Microarray analysis: changes in gene expression caused by chronic CTR deficiency

Since long-term CTRKO genotype produced a remarkable reversal of T-antigen-induced prostate carcinogenesis in LPB-Tag mice, we examined differential gene expression among experimental groups using RNA extracted from the prostates on Affymetrix Clariom D™ mice microarrays. Gene expression profiles of prostates of WT, LPB-tag, CTRKO and CTRKO-LPB-Tag genotypes were generated using the Clarion D Pico Assay mouse array that represents > 66,100 mouse genes and > 214,900 transcripts.

**Figure 3:**
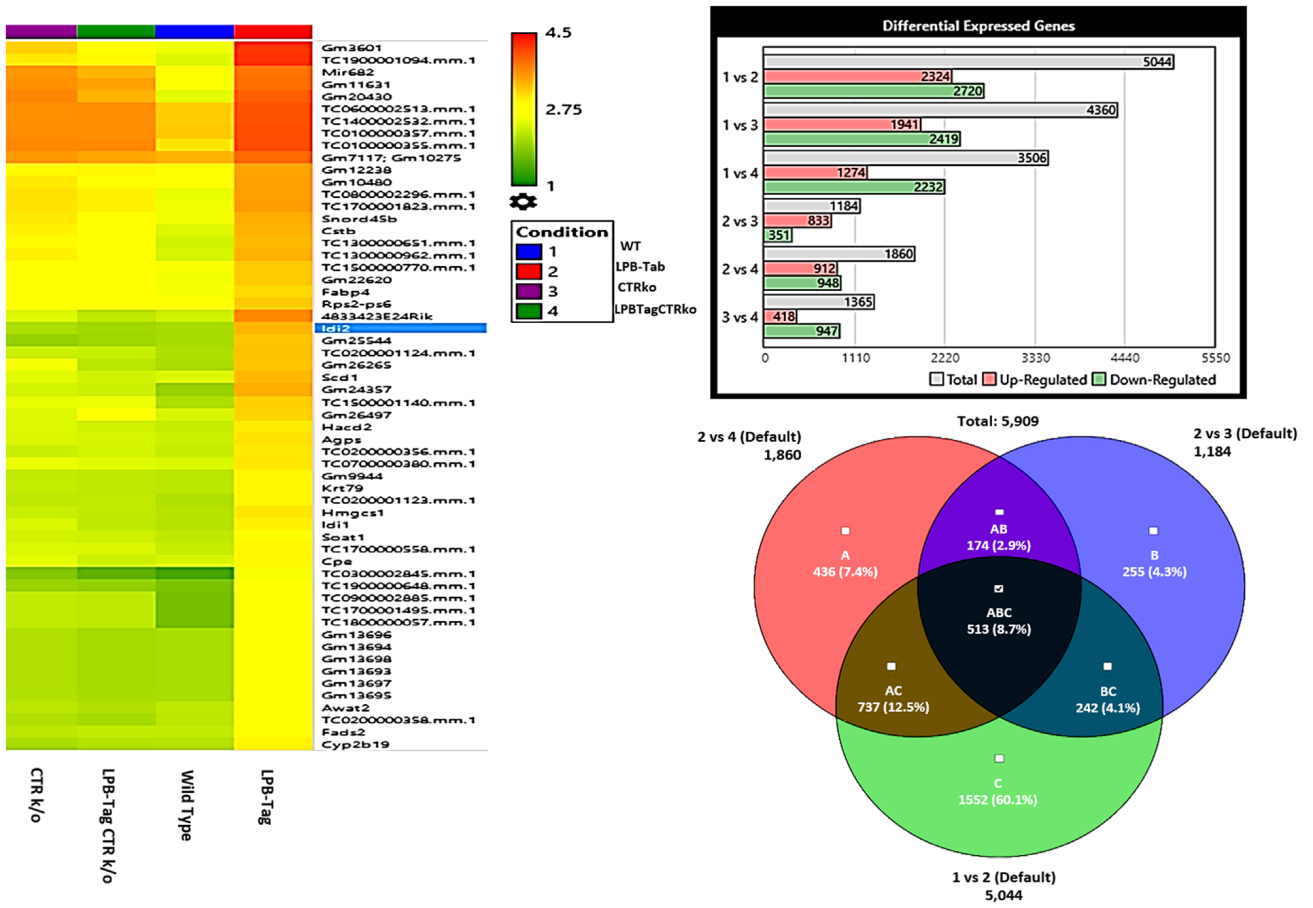
Microarray analysis. Figure represents hierarchical clustering of genetic data, quantitative representation of differentially expressed genes, and interrelated genetic expression Venn diagram for the rna samples extracted from WT, LPB-Tag, CTRKO, and LPB-Tag-CTRKO mice. Columns represent samples and rows represent genes. The bar graph shows intergroup comparison of the number of genes expressed differentially in WT, LPB-Tag, CTRKO, and LPB-Tag-CTRKO mice.

**Figure 4:**
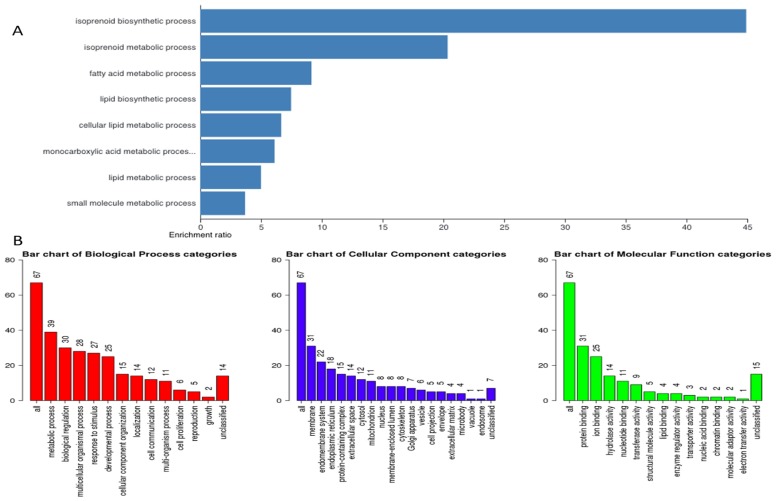
Gene ontology. (**A**) Representative figure depicts gene enrichment and over representation of 67 genes in the gene ontology extracted important pathways representing different biological processes, cellular component, and molecular function. (**B**) Representative figure shows differential involvement of these 67 genes of interest in different biological processes. Larger number represents higher gene involvement in that biological process.

Next, we hypothesized that filtering tumor-associated genes of LPB-Tag genotype against the other three prostate groups with normal prostate phenotypes will help identify clusters of genes associated with prostate carcinogenesis. Venn analysis of the gene expression data identified 948 down-regulated and 912 up-regulated genes (Figure 3). Of these top 67 were most overexpressed genes in prostate tumors across the meta-analysis (FDR, V5%, Supplementary Table 1). We performed pathway analysis using Transcriptome Analysis Console (TAC) 4.0 (ThermoFisher Scientific), WEB-based GEne SeT AnaLysis Toolkit (WebGestalt. org) and ShinyGO V0.51 online software (sdstate. edu). The resulting datasets of all four experimental groups were analyzed for differences in the gene expression and associated pathways. A total of eight pathway networks were generated. The first four networks with scores of 19, 18, 18, and 12 indicated that genes within these pathways regulated small molecule metabolic process, lipid metabolic process, cellular metabolic process, and lipid biosynthetic process respectively (Figure 4A, 4B). High scores of these networks suggest that they were generated by including the data of larger number of CT-regulated genes, thus raising the probability that CT regulates prostate tumor growth and metastasis through one or more of these networks.

### Hierarchical clustering and gene ontology

To visualize the array results, we performed hierarchical clustering of most significantly altered genes. Several of these genes displayed many-fold decreased expression in the LPB-Tag-CTRKO genotype as compared to LPB-Tag genotype (Figure 3). For example, clustered genes like Scd1, Fabp4, HMGCS1, and Agps. To further elucidate the functions of these clustered genes, GO analysis was performed using a multiple test adjustment method based on R function and Bonferroni test with a significance level of *P* ≤ 0.05. In terms of biological process, the increased gene expression in LPB-Tag group showed an over-representation of several important genes of metabolic activities (Supplementary Table 1), including the isoprenoid, steroid, and lipid biosynthesis (Table 1). In particular, genes encoding HMG-CoA synthase, HMG-methyl CoA reductase, and steroyl CoA desaturase were highly up-regulated in the tumors of LPB-Tag mice but were underexpressed in the prostates of LPB-TagCTRKO as well as CTRKO genotypes, suggesting that CTR may be a key enhancer of tumor associated lipid metabolism and hypercholesterolemia.

**Table 1:** Representative list of genes involved in lipid metabolism, adipogenesis, and triglyceride synthesis showing differential expression in LPB-Tag group as compared to CTRKO and LPB-Tag-CTRKO groups

Lipid Metabolism			
Gene of Interest	LPBTag vs WT (Fold Change)	LPBTag vs CTRk/o (Fold Change)	LPBTag vs LPBTagCTRk/o (Fold Change)
3-Hydroxy-3-methylglutaryl-CoA-Synthase1 (Hmgcs1)	12.82	10.79	9.46
3-Hydroxy-3-methylglutgaryl-CoA-Reductase (Hmgcr)	1.98	1.88	1.86
Isopentenyl Diphosphate delta isoform (ldi1)	9.67	5.64	8.55
Stearoyl CoA Desaturase (Scd1)	24.62	21.67	28.4
Fatty Acid Desaturase 2 (Fads2)	5.12	5.75	5.12
Fatty Acid Desaturase 1 (Fads1)	2.25	2.13	2.26
Sterol O-Acetyl Transferase 1 (Soat1)	6.75	5.2	5.52
Acyl CoA Synthetase Long Chain Member 1 (Acsl1)	3.53	2.62	3.1
**Adipogenesis**			
Fatty Acid Binding Protein 4 (Fabp4)	6.55	4.38	5.32
Stearoyl CoA Desaturase 1 (Scd1)	24.62	21.67	28.4
**Triglyceride Synthesis**			
Alkylglycerone Phosphate Synthase (Agps)	10.12	8.2	8.82

Gene level analysis *p*-value was < 0.05; Gene level FDR was set to < 0.05.

Next, we hypothesized that filtering the CT-regulated genes in WT against CTRKO genotypes and LPB-Tag against LPB-Tag-CTRKO sample set would be an ideal approach. CTRKO genotype led to significant changes in the expression of several genes (Supplementary Table 1). Since some of these genes displayed several-fold change, they could represent novel targets of CT-induced prostate carcinogenesis. We classified CT-regulated genes into two, cancer-related and androgen-responsive sub groups as depicted in Table 2. Several known cancer-related genes including prolactin-inducing protein, Heat shock protein 40 homolog, chemokine (C-C motif) receptor 7, programmed cell death 4 as well as other genes like retinoblastoma1 (Rb), phosphate and tensin homolog (PTEN), early growth response 2 (Egr2), protocadherin 4, and RNA binding motif protein 4 displayed remarkably lower expression in the prostates of WT and LPB-Tag genotypes as compared to their corresponding CTRKO genotypes (For example, WT vs CTRKO; LPB-Tag vs LPB-TagCTRKO. In contrast, metastasis-promoting genes such as prostate stem cell antigen (PSCA), matrix metallopeptidase 7, and CD44 standard antigen displayed significantly higher expression in WT and LPB-Tag groups as compared to their corresponding CTRKO groups. Also, inflammation related genes like cytochrome p450 and catalase showed relatively higher expression in the prostates of WT and LPB-Tag while immune function related genes like transducer erbB-2.1 showed relatively lower expression (Table 2). These results provide some insights into the mechanisms associated with the role of CTR in promoting prostate carcinogenesis.

**Table 2:** Representative list of genes related to cancer, metastasis, inflammation, immune function, cell-matrix interaction, and androgen function, showing differential expression in WT vs CTRKO groups and LPB-Tag vs LPB-Tag-CTRKO groups

Gene	WT (CT+) expression as compared to CTR-ko (CT-) Log2 (Fold change)	LPB-Tag (CT+) expression as compared to LPB-Tag-CTR-ko (CT-) Log 2 (Fold Change)
**Cancer Related**		
**Cystatin B (CstB)**	7.68	8.96
**Prostate stem cell antigen (PSCA)**	3.86	2.16
**Enoyl Coenzyme A hydratase domain containing 1 (Echdc1)**	1.83	2.11
**Neural precursor cell expressed, developmentally down-regulated 4 (Nedd4)**	1.71	1.98
**Claudin 4 (Cldn4)**	1.68	1.87
**Fibronectin 1 (Fn1)**	1.42	1.52
**Regulator of G-protein signalling 21 (Rgs21)**	1.42	1.52
**Member RAS oncogene family (Rab25)**	1.31	1.55
**Prosaposin (Psap)**	1.22	1.78
**DNA-damage inducible transcript 3 (Ddit3)**	−1.29	−1.58
**BCL2-like 14 (Bcl2l14)**	−1.3	−1.53
**RNA binding motif protein 4 (Rbm4)**	−1.31	−1.51
**Retinoblastoma 1 (Rb1)**	−1.37	−1.68
**Early growth response 2 (Egr2)**	−1.4	−1.57
**Phosphate and tensin homolog (Pten)**	−1.42	−1.54
**Protocadherin beta 4 (Pcdhb4)**	−3.62	−2.71
**DnaJ10 (Hsp40) homolog subfamily member 10**	−4.35	−3.65
**Prolactin Induced Protein (Pip)**	−7.94	−3.79
**Metastasis Related**		
**Matrix metallopeptidase 7 (Mmp7)**	1.74	2.45
**CD44 antigen (CD44) standard**	1.44	1.56
**Transmembrane protease, serine 2 (Tmprss2)**	1.24	1.69
**Mitogen-activated protein kinase 3 (Mapk3)**	1.24	1.51
**NME/NM23 nucleoside diphosphate kinase 2 (Nme2)**	−1.37	−1.55
**Inflammation Related**		
**Cytochrome P450, family 2, subfamily e, polypeptide 1 (Cyp2e1)**	3.32	3.67
**Catalase (Cat)**	1.64	1.77
**Pyruvate dehydrogenase kinase, isoenzyme 4 (Pdk4)**	1.29	2.43
**Caspase 4 (Casp4)**	−1.12	−1.57
**Interleukin 4 receptor, alpha (IL4ra)**	−1.17	−1.52
**Caspase 1 (Casp1)**	−1.34	−1.52
**Erythroid differentiation regulator 1 (Erdr1)**	−1.43	−1.74
**Immune Function Related**		
**Cytochrome c oxidase subunit II (Cox2)**	1.91	2.31
**CD52 antigen (CD52) immune function**	1.77	2.26
**Transducer of ErbB-2.1 (Tob1)**	−1.7	−1.84
**Cell-Matrix Interaction Related**		
**Dermatopontin (Dpt)**	3.44	3.66
**Androgen Related**		
**Heat shock protein 90, beta member 1 (Hsp90b1)**	3.12	3.62
**Defensin beta 34 (Defb34)**	2.95	3.18
**Hepatoma-derived growth factor (Hdgf)**	2.46	2.45
**Calnexin (Canx)**	2.44	2.33
**Alcohol dehydrogenase 1 (class I) (Adh1)**	2.40	2.53
**CD24a antigen (cd24a)**	2.23	2.13
**Ezrin (Ezr)**	2.13	2.74
**Transglutaminase 4 (prostate) (Tgm4)**	2.08	2.55
**Serine/arginine repetitive matrix 2 (Srrm2)**	−2.16	−2.41
**TRPM8 channel-associated factor 3 (Tcaf3)**	−2.24	−2.81
**Programmed cell death 4 (Pdcd4)**	−2.57	−2.54
**Decorin (Dcn)**	−2.68	−2.77
**Gasdermin C3 (Gsdmc3)**	−2.83	−2.51
**Chemokine (C-C motif) receptor 7 (Ccr7)**	−3.24	−2.13
**NADH dehydrogenase subunit 4 (ND4)**	−3.45	−2.73

Positive values represent increased expression while negative values represent decreased expression in WT and LPB-Tag groups in comparison to CTRKO and LPB-Tag-CTRKO groups respectively. Gene level analysis *p*-value was < 0.05; Gene level FDR was set to < 0.05.

**Figure 5:**
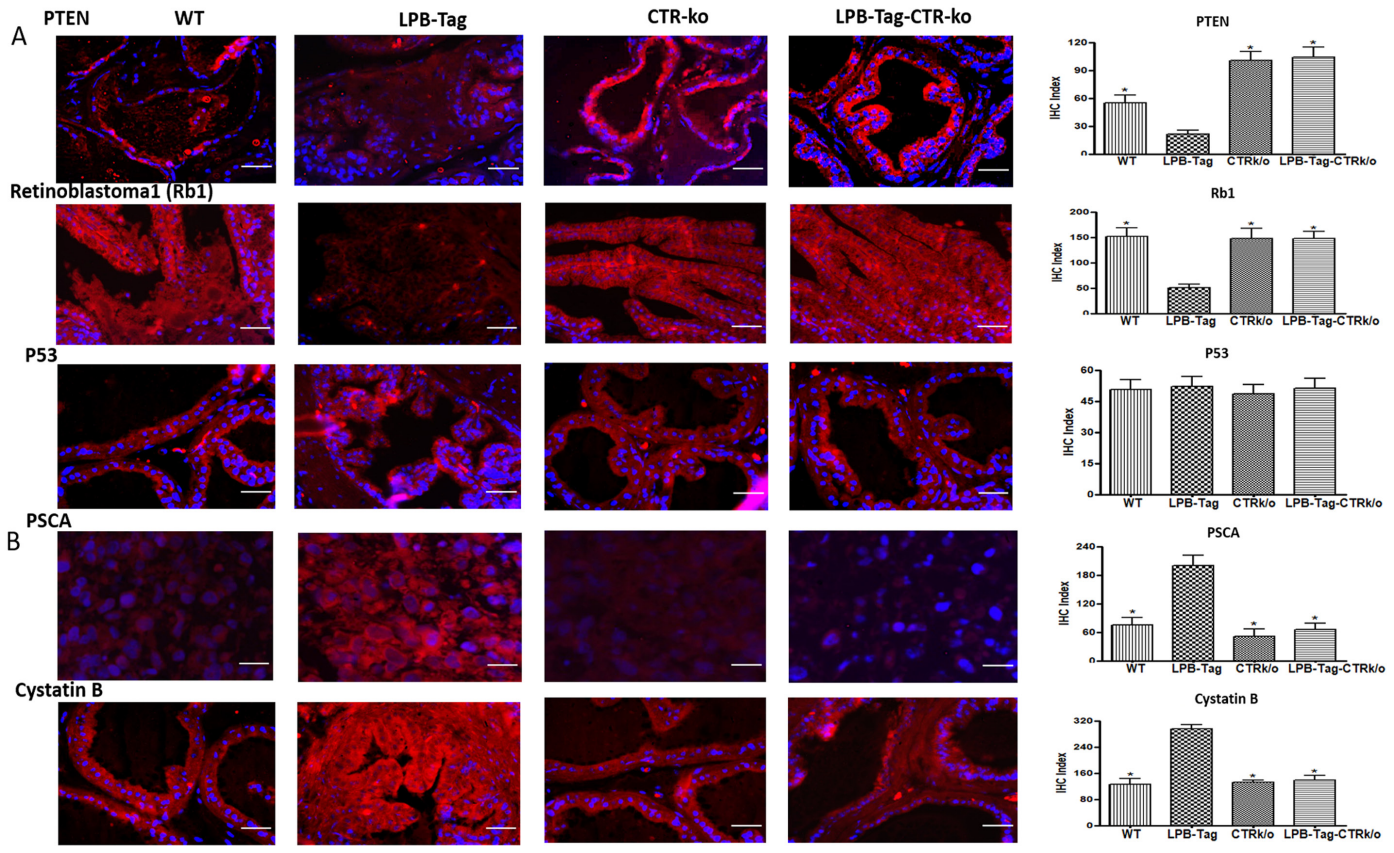
Immunoreactivity of tumor suppressor genes. (**A**) Representative photomicrographs and corresponding IHC index graphical representation of the PTEN, Rb1, and p53 immunofluorescence in the prostate tissue of WT, CTRKO, LPB-Tag, and LPB-Tag-CTRKO mice. The red staining panels represent PTEN, Rb1, or p53 activity respectively while blue staining represents the DAPI staining at 40× magnification. ^*^ represents significantly different than LPB-Tag; *p* < 0.05; Scale bar 100 µm. (**B**) Representative photomicrographs and corresponding IHC index graphical representation of the PSCA and cystatin b immunofluorescence in the prostate tissue of WT, CTRKO, LPB-Tag, and LPB-Tag-CTRKO mice. The red staining panels represent PSCA or cystatin b activity respectively while blue staining represents the DAPI staining at 40× magnification. ^*^ represents significantly different than LPB-Tag; *p* < 0.05; Scale bar 100 µm.

Signature tumor suppressor genes of prostate cancer such as Rb, PTEN, Caspase 1, and Tob-1 were underexpressed in LPB-Tag tumors but were overexpressed in the prostates of LPB-TagCTRKO genotype. These findings were subsequently confirmed by qRT-PCR and immunofluorescence studies of PTEN and Rb1 proteins in the prostate sections (Figure 5A). Interestingly, p53 was not significantly affected when assessed by microarray analysis as well as by immunofluorescence. In contrast, signature tumor promoting PSCA gene was overexpressed in LPB-Tag mice but was underexpressed in CTRKO-LPBTag as well as CTRKO mice (Figure 5B). Similarly, cancer associated cystatin b gene was overexpressed in LPB-Tag mice as compared to CTRKO-LPBTag as well as CTRKO mice (Figure 5B).

### CTRKO genotype displayed remarkable changes in the expression of androgen-regulated genes

Since CTR has been reported to mimic some actions of androgens on prostate cells [13], we directed our attention to androgen-regulated genes that displayed significant changes in their expression based on the presence/absence of CTR in their prostates. We identified the genes that displayed 1.5-fold or greater difference in their expression in the prostates of CTRKO/LPBTag-CTRKO mice as compared to their corresponding CTR-positive counterparts, WT, and LPBTag (Table 2). We then selected genes associated with cancer such as Prolactin-inducing protein (8-fold greater expression in CTRKO prostates as compared to WT, 4-fold greater in LPB-Tag-CTRKO as compared to LPB-Tag). Similarly, the expression of several other genes such as HSP40 homolog, heat shock protein 90, defensin beta 34, and prostate transglutaminase 4 was also several-fold higher than CTRKO groups as compared to their WT or LPB-Tag groups. In contrast, NADH dehydrogenase subunit 4, programmed cell death 4, and TRPM8 channel associated factor 3 showed relatively lower expression in CTRKO groups suggesting a role of CTR in regulation of activity of these genes (Table 2).

### Quantitative real-time PCR

**Figure 6:**
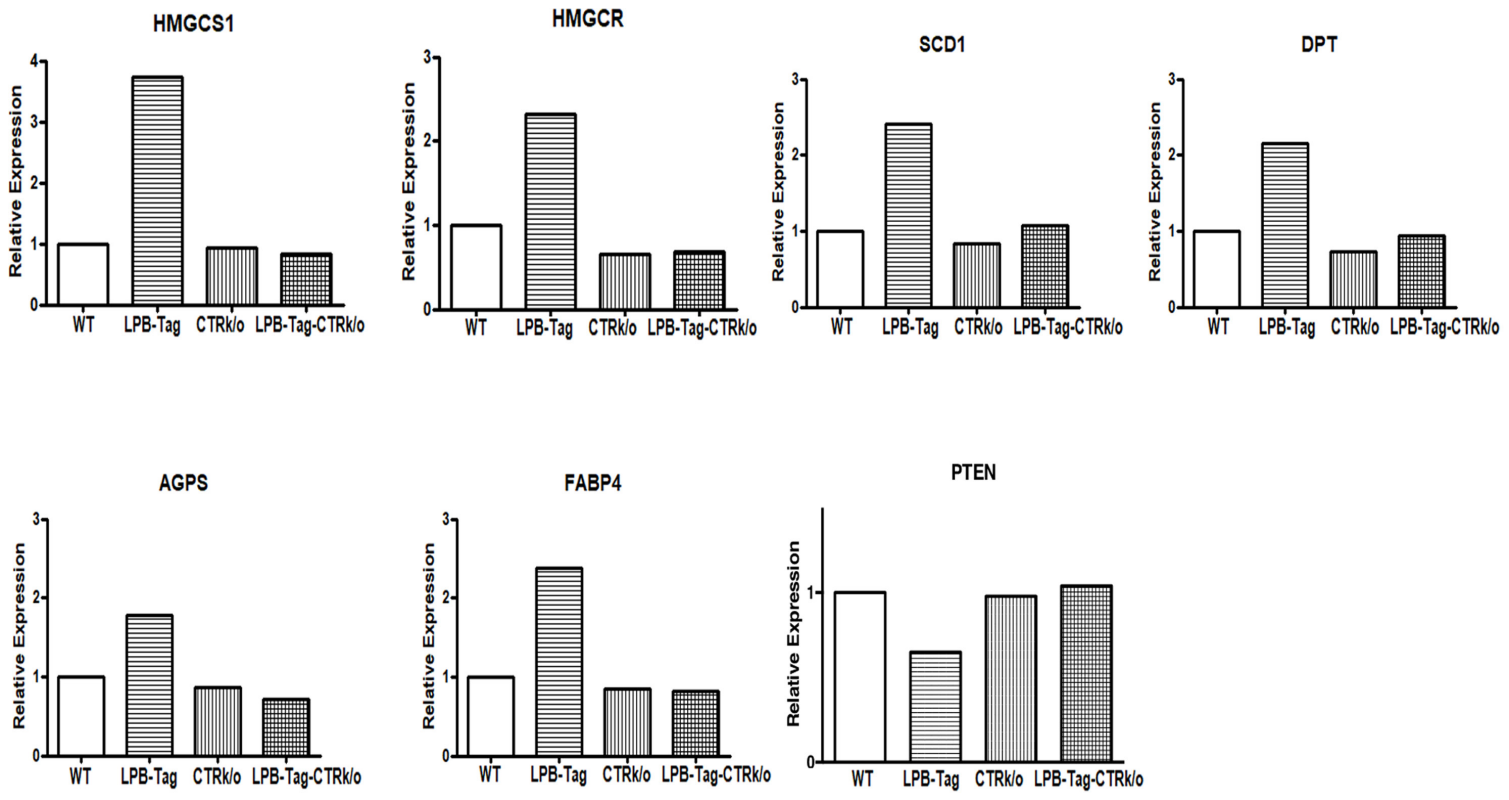
Gene expression of different genes of interest. Figure represents relative gene expression by quantitative RT-PCR of important microarray data extracted genes, HMGCS1, HMGCR, SCD1, DPT, AGPS, FABP4, and PTEN to validate microarray results in the WT, CTRKO, LPB-Tag, and LPB-Tag-CTRKO groups.

We verified CTR-associated changes in the expression profile of some important cancer-associated genes by qRT-PCR. Aliquots of cDNA that were used for microarray analysis were also used for qRT-PCR. The results showed a close stringency with the array result with changes in expression of the genes HMGCS1, HMGCSR, SCD1, DPT, AGPS, FABP4, and PTEN respectively (Figure 6). GAPDH was used as a positive control. Consistent with the microarray data, we found a higher expression of HMGCS1 (3.5-fold), HMGCR (2.5-fold), SCD1 (2.5-fold), AGPS (1.9-fold), FABP4 (2.5-fold), DPT (2.2- fold), and PTEN (1.56-fold decline) in prostate tumors of LPB-Tag genotype when compared with the prostates of other genotypes. Among these genes, PTEN is an important tumor suppressor gene predominantly involved in human prostate tumors, whereas HMGCS1, HMGCR, SCD1, FABP4, and SCD1are critical enzymes in the pathways of lipid synthesis as well cholesterol and steroid synthesis. Dermatopontin (DPT) is an extracellular matrix protein with possible functions in cell-matrix interactions and matrix assembly.

### Prolonged CTR deficiency induces epithelial markers but suppresses mesenchymal markers in LPB-Tag mouse prostates

**Figure 7:**
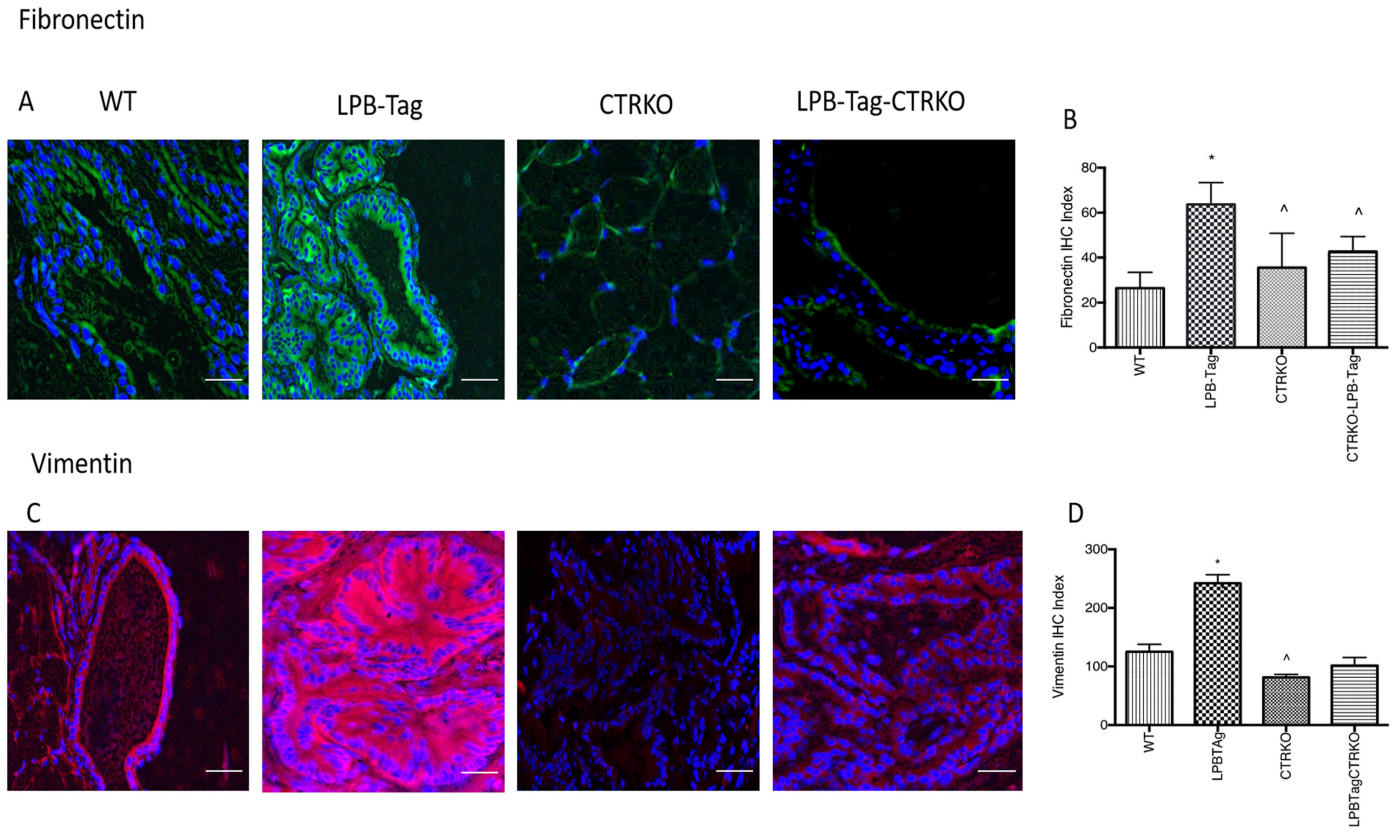
Immunoreactivity of fibronectin and vimentin. (**A**) Representative photomicrographs of immunofluorescence for fibronectin in the prostate tissues of WT, CTRKO, LPB-Tag and LPB-Tag-CTRKO mice. Green staining represents fibronectin activity while blue staining represents the DAPI at 40× magnification; Scale bar 100 µm. (**B**) Figure represents mean IHC staining index for the fibronectin immunofluorescence observed in the prostate tissues of WT, CTRKO, LPB-Tag and LPB-Tag-CTRKO mice; ^*^ represents significantly different than WT and ^^^ represents significantly different than LPB-Tag; *p* < 0.05. (**C**) Representative photomicrographs of immunofluorescence for vimentin in the prostate tissues of WT, CTRKO, LPB-Tag and LPB-Tag-CTRKO mice. Red staining represents vimentin activity while blue staining represents the DAPI at 40× magnification; Scale bar 100 µm. (**D**) Figure represents mean IHC staining index for the vimentin immunofluorescence observed in the prostate tissues of WT, CTRKO, LPB-Tag and LPB-Tag-CTRKO mice; ^*^ represents significantly different than WT and ^^^ represents significantly different than LPB-Tag; *p* < 0.05.

**Figure 8:**
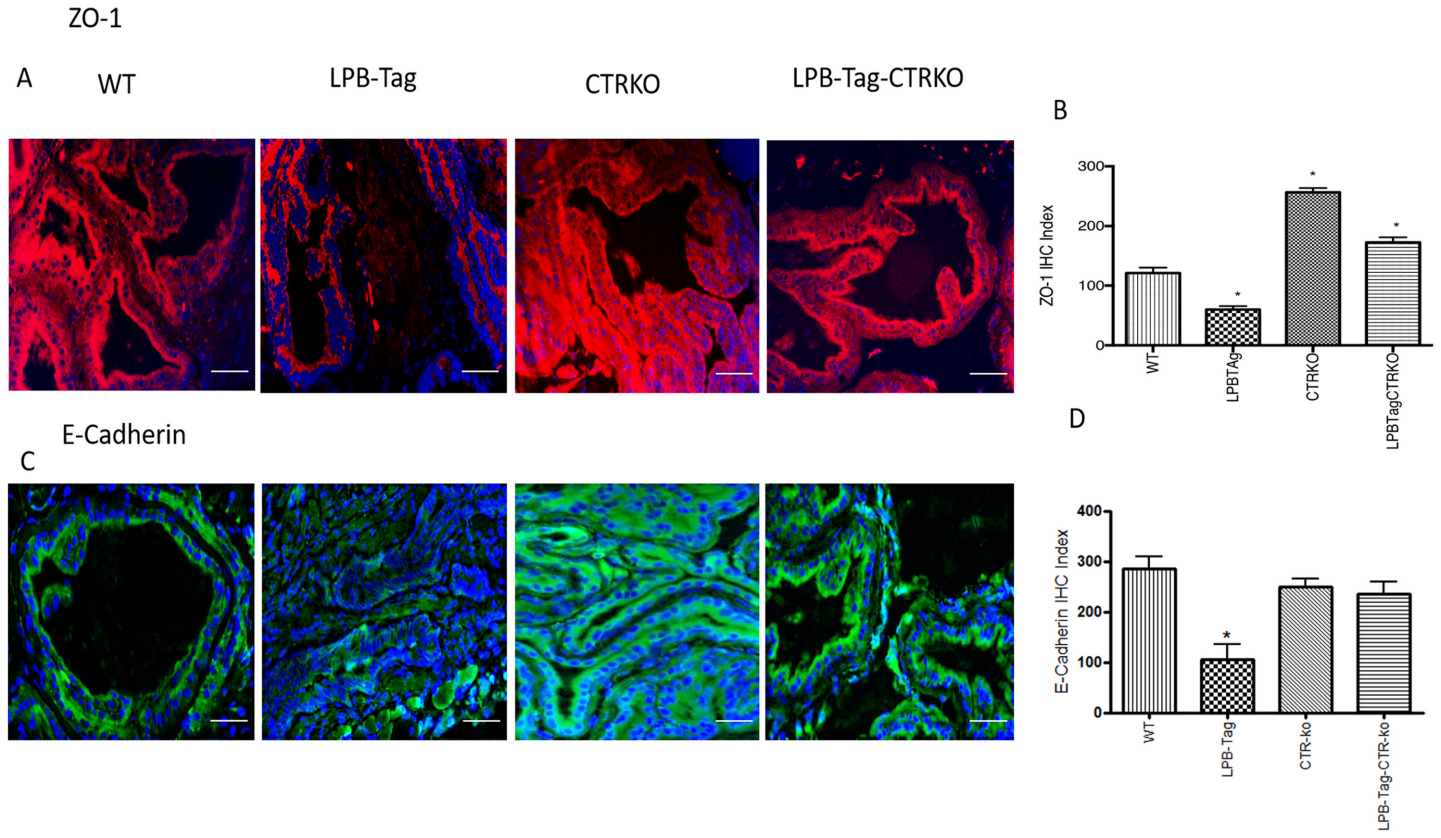
Immunoreactivity of ZO-1 and E-cadherin. (**A**) Representative photomicrographs of immunofluorescence for ZO-1 in the prostate tissues of WT, CTRKO, LPB-Tag and LPB-Tag-CTRKO mice. Red staining represents fibronectin activity while blue staining represents the DAPI at 40× magnification; Scale bar 100 µm. (**B**) Figure represents mean IHC staining index for the ZO-1 immunofluorescence observed in the prostate tissues of WT, CTRKO, LPB-Tag and LPB-Tag-CTRKO mice; ^*^ represents significantly different than WT; *p* < 0.05. (**C**) Representative photomicrographs of immunofluorescence for E-cadherin in the prostate tissues of WT, CTRKO, LPB-Tag and LPB-Tag-CTRKO mice. Green staining represents vimentin activity while blue staining represents the DAPI at 40× magnification; Scale bar 100µm. (**D**) Figure represents mean IHC staining index for the E-cadherin immunofluorescence observed in the prostate tissues of WT, CTRKO, LPB-Tag and LPB-Tag-CTRKO mice; * represents LPB-Tag significantly different than WT, CTRKO, LPB-Tag-CTRKO; *p* < 0.05.

**Figure 9:**
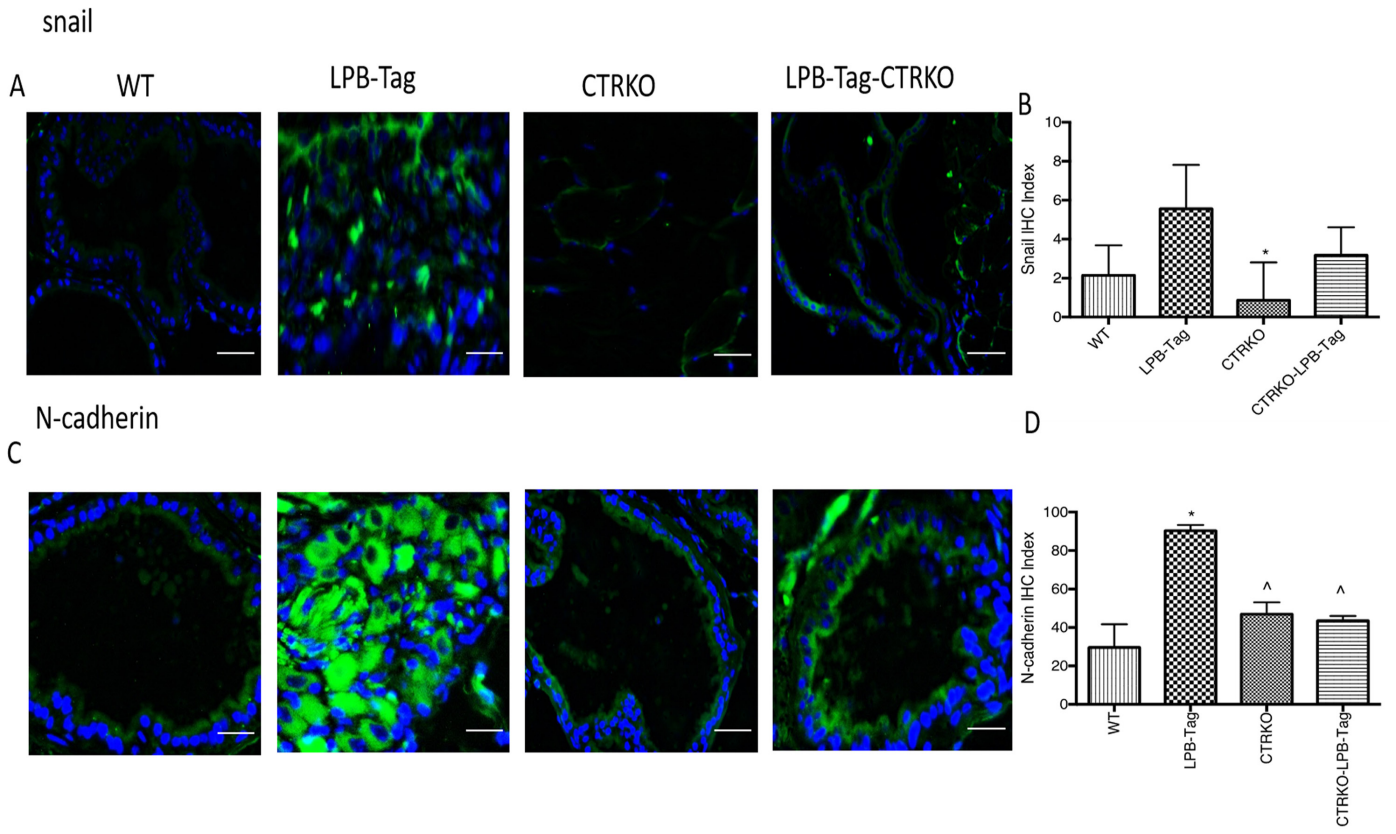
Immunoreactivity of snail and N-cadherin. (**A**) Representative photomicrographs of immunofluorescence for snail in the prostate tissues of WT, CTRKO, LPB-Tag and LPB-Tag-CTRKO mice. Green staining represents snail activity while blue staining represents the DAPI at 40× magnification; Scale bar 100 µm. (**B**) Figure represents mean IHC staining index for snail immunofluorescence observed in the prostate tissues of WT, CTRKO, LPB-Tag and LPB-Tag-CTRKO mice; ^*^ represents significantly different than LPB-Tag; *p* < 0.05. (**C**) Representative photomicrographs of immunofluorescence for N-cadherin in the prostate tissues of WT, CTRKO, LPB-Tag and LPB-Tag-CTRKO mice. Green staining represents N-cadherin activity while blue staining represents the DAPI at 40× magnification; Scale bar 100 µm. (**D**) Figure represents mean IHC staining index for the N-cadherin immunofluorescence observed in the prostate tissues of WT, CTRKO, LPB-Tag and LPB-Tag-CTRKO mice; ^*^represents significantly different than WT and ^^^ represents significantly different than LPB-Tag; *p* < 0.05.

Since our earlier studies have shown that CTR induced EMT in prostate cancer cells, we examined the effect of CTRKO on EMT markers in mouse prostates of all groups [13]. LPB-Tag mice displayed elevated expression of mesenchymal markers fibronectin and vimentin (Figure 7), but diminished expression of epithelial markers ZO-1 and E-cadherin (Figure 8). EMT markers Snail and N-cadherin also followed a similar pattern suggesting that CTRKO abolishes T-antigen-induced EMT in the prostates of LPB-Tag mice (Figure 9).

## DISCUSSION

Earlier studies from this laboratory have shown that the activated CT-CTR axis is a robust promoter of growth and invasiveness of multiple PC–derived cell lines in culture as well as *in vivo* when PC-derived cell lines are orthotopically implanted in immunodeficient mice [8, 10, 13, 16]. The present study examined the role of CTR in the early prostate oncogenesis in LPB-Tag prostate cancer mouse model. Histology of the LPB-Tag mouse prostates indicated the prevalence of proliferative overgrowth, dysplasia, and adenocarcinoma. Although the precise mechanism by which Tag induces prostate carcinogenesis in B6 mice is not known, the current evidence suggests that oncogenic *Tag* induces prostate neoplasia by sequestering and inactivating two tumor suppressor genes, *p53* and *RB, t*hus accelerating cell cycle progression for rapid cell proliferation [17]. This enables prostatic epithelial cells to proliferate rapidly and form PIN lesions, but this mechanism may not be sufficient to form fully developed PC. These pre-cancerous cells generally need to acquire a second mutation or “hit” to acquire the ability to invade into the surrounding stromal tissue and form malignant PC [15, 18]. The present results that T-antigen expression in the prostate led to the suppression of PTEN gene expression may provide a second hit and contribute to malignancy.

The present studies demonstrated that CTRKO genotype abolished Tag-induced carcinogenesis. Interestingly, this robust effect of CTR deficiency on carcinogenesis was observed even in the presence of T-antigen expression in LPB-Tag-CTRKO mice, suggesting that CTR alters critical pathway (s) associated with T-antigen-induced carcinogenesis. Histological evidence of tumor absence in these mice was further corroborated by the results that the prostates of these mice displayed greatly reduced number of Ki67-positive cells as compared to those of LPB-Tag mice. Our earlier studies have shown that CTR increased growth and metastatic characteristics of the prostate cancer cells by multiple actions that included disruption of cell-cell junctions and activation of Gαs-mediated Wnt-β-catenin and PI3Kinase-Akt-survivin signaling pathways [11, 13]. It is conceivable that the deficiency of CTR attenuated prostate carcinogenesis by modulating either or all of these mechanisms. Differential gene expression data of these prostates revealed that the CTRKO genotype altered multiple signaling pathways when compared with WT as well as with LPB-Tag mice. For example, there was a significant increase in Rb gene expression in CTRKO/LPB-Tag-CTRKO genotypes as compared to WT and LPB-Tag genotypes. Moreover, PTEN suppression was observed in LPB-Tag genotypes, but not in CTRKO-LPB-Tag prostates, suggesting for the first time that CTR may facilitate PTEN silencing in LPB-Tag mice. PTEN is a lipid phosphatase, which regulates the apoptosis and cell migration and adhesion pathways by negatively regulating the function of proteins such as Akt [19, 20]. In the absence of PTEN, activated Akt levels remain elevated leading to the activation of survival pathways [21]. In addition to antiapoptotic effect, PTEN directly dephosphorylates FAK and Shc, which leads to suppression of cell migration, adhesion, and invasion. Loss of PTEN activity promotes the detachment of cells from the extracellular matrix and increases cell migration and invasion [22]. Considering the important role of PTEN silencing/mutations in human prostate cancers [23], these results were positively verified by qRT-PCR as well as immunofluorescence. Our earlier reports that anti-apoptotic activity of CT in prostate cancer cells can be reversed by specific PI3K inhibitors are consistent with our present results [11]. The present results that CTRKO genotype down-regulated PSCA expression in prostates as compared to their CTR-positive counterparts are also consistent with the evidence that PTEN–regulated signaling pathways cause cancer and up-regulate the expression of PSCA [24]. Additional studies are underway to examine the role of the CT-CTR axis in PTEN regulation of the prostate.

The present studies have for the first time identified that T-Ag-induced prostate carcinogenesis is associated with overexpression of several enzymes of lipid metabolism, especially those involved in synthesis of fatty acids (FAs), phospholipids, and cholesterol. However, the presence of CTRKO genotype abolished all Tag-induced changes in lipid metabolism of the prostate including those involving synthesis of lipogenesis, cholesterol biosynthesis, and lipid desaturation suggesting that CTR signaling may have an important role in the induction of genes associated with lipid metabolism. The present results for the first time suggest that CTR may have direct effect on up-regulating lipid metabolism in the prostate gland; and are consistent with a recent report that CTRKO mice exhibited weight loss when 15 months old with significant decrease in liver, adipose tissue, and kidney weights compared with wild-type control mice [25].

T-Ag-induced enzymes may up-regulate de novo FA synthesis, which is crucial for tumors [26]. Newly synthesized FAs can then be esterified with glycerol to generate triacylglycerols (TGs) or sterol esters (SEs), respectively, and then stored in lipid droplets (LDs). These FAs may then be used either for incorporation into membrane, storage, synthesis of signaling lipid molecules, or for energy. The LPB-Tag prostates displayed several-fold increase in the expression of several enzymes associated with lipid biosynthetic pathway such as SCD1, ASCL1, HMG-CoA synthase and reductase (HMGCS and HMGCR, respectively) [27, 28]. Cholesterol is an important membrane component because it modulates the fluidity of the lipid bilayer and provides the structural backbone for the synthesis of steroid hormones such as testosterone [28]. Hypercholesterolemia is associated with prostate cancer, and statins are effective in reducing the risk of prostate cancer, and dysregulation of cholesterol metabolism at the cellular level [29, 30]. It has been recently suggested that cholesteryl ester accumulation in lipid droplets within prostate cancer cells is a causative factor underlying prostate cancer aggressiveness [31]. This aberrant accumulation was observed only in high grade and metastatic disease and is absent in normal tissue, benign prostatic hypertrophy, prostatitis, and prostatic intraepithelial neoplasia. Loss of PTEN and subsequent activation of PI3K/Akt/ mTOR with upregulation of SREBF and LDL receptors was shown to induce cholesteryl ester accumulation but was independent of androgen signaling. When esterification was blocked at the level of the acetyl-coA cholesterol acyltransferase enzyme, either pharmacologically or by siRNA treatment, there was an observed increase in apoptosis accompanied by a decrease in cellular proliferation, migration, and invasion both *in vitro* and *in vivo* [31]. In addition, cholesterol and other membrane lipids are required to form cholesterol-rich membrane rafts. These specialized structures play an important role in membrane trafficking and assembling of signaling complexes on the plasma membrane [32]. Lipids are also important signaling molecules. Phosphoinositides are important second messengers that relay signals from activated growth factor receptors to the cellular machinery. Their actions are critical for the recruitment of effector proteins to specific membrane compartments. One of the most prominent lipids of this class is phosphatidylinositol (3,4,5)-trisphosphate [PtdIns (3,4,5)*P*_3_; PIP_3]_. This molecule is produced by PI3K in response to the growth factor signaling and mediates the recruitment and activation of Akt. PIP_3_ is also the substrate for PTEN [33, 34].

We also identified multiple prostate cancer-associated genes such as cystatin C, PSCA, Cyp2e1, and dermatopontin that were remarkably underexpressed in CTRKO/LPB-Tag-CTRKO as compared to their WT and LPB-Tag counterparts, suggesting that CTR positively regulates these genes. Cystatin C is an important inhibitor of cathepsin B and tumor cell invasion [35, 36]. Cathepsin B influences tumor microenvironment by degradation of extracellular matrix and by activation of other proteolytic enzymes such as pro-urokinase-type plasminogen activator (pro-uPA) and matrix metalloproteases (MMPs) so that tumor cells can actively invade and metastasize [37]. Cystatin C has been suggested to play an important role in the neuroendocrine differentiation of prostate cancer [38]. More recently, serum cystatin C has been proposed as a useful marker of increased osteoblastic activity associated to bisphosphonate treatments in prostate cancer patients with bone metastasis [39]. Although a direct link between cystatin C and extracellular matrix protein MMP2 in prostate cancer has been suggested, a precise role of cystatin C in prostate cancer progression has not been established [40]. Cyp2e1 is involved in the processes associated with inflammation, whereas dermatopontin plays an important role in cell-matrix interactions and matrix assembly, and modulates the behavior of TGF-β to support the induction of EMT [41, 42]. We also identified several cancer-related genes such as protocadherin β4, HSP40 homolog, and prolactin-induced protein (PIP) that were overexpressed in CTRKO/LPB-Tag-CTRKO as compared to WT and LPB-Tag counterparts, suggesting CTR downregulates these genes. PIP is a small secreted glycoprotein carrying several N-linked carbohydrate chains. The expression of PIP is generally restricted to cells with apocrine properties [43]. Being a secretory protein, PIP is present in seminal plasma, saliva, lacrimal fluid, tears, and sweat gland secretion. Little is known about the biological role of PIP. PIP plays an important role in cell-mediated adaptive immunity and binds to bacteria from several genera, which suggests that this glycoprotein may participate in innate immunity and protection of hosts against microbial infections. Increased levels of PIP were found in several types of human cancer such as prostate, sweat, and salivary gland cancers.

Considering the importance of AR in the prostate cancer, we also examined whether CTRKO genotype alters the expression of AR-regulated genes. CTRKO phenotype caused a remarkable downregulation of HSP90β1, Defensin β34, Hepatoma-derived growth factor, Calnexin, CD24α antigen, and Ezrin genes but caused up-regulation of programmed cell death 4 gene. HSP90 is the important chaperone for AR and its downregulation in CTRKO genotype suggests that CTR may play an important role in HSP90 levels, thereby affecting the transport of AR to the nucleus and subsequent biological response of androgen action [44–46]. Decreased expression of HDGF may support decreased proliferation, whereas decreased EZRIN expression may attenuate cell adhesion [47, 48]. Defensin and CD24 are immune proteins associated with inflammatory response. We observed induction of HSP90 gene in CTRKO genotype. Whether other actions of CTRKO genotype occur prior to or subsequent to the induction of HSP90 gene remains to be investigated. Also, CTRKO genotype induced the expression of PCDC4, a marker of apoptosis, further confirming tumor-suppressing properties of CTRKO genotype [49].

In conclusion, the results of the present study indicate that CTRKO genotype inhibits T-antigen-induced prostate carcinogenesis in LPB-Tag mice without causing any apparent harmful side effects or reducing T-antigen expression. Considering the similarity in the actions of CT-CTR axis and T-antigen on prostate cancer cells such as the activation of Wnt/β-catenin, a PI-3-kinase-Akt-survivin signaling and the importance of these pathways in carcinogenesis, EMT, and acquisition of stem cell phenotype, it is likely that the presence of CTR may be critical for activation of these pathways. Moreover, the study has identified several targets of CTR that may play an important role in prostate carcinogenesis and includes PTEN, Rb as well as several genes associated with lipid metabolism, androgen receptor transport, cell proliferation, adhesion, inflammation, and immune function.

## MATERIALS AND METHODS

### Generating/genotyping transgenic mice

#### CTRKO mice

Founder heterozygous calcitonin receptor knockout mice (two males and two females) were purchased from the Mutant Mouse Regional Resource Center (MMRRC) at the University of North Carolina, Chapel Hill (RRID: MMRRC 011609-UNC).

#### LPB-Tag transgenic mice

The founder *Lady* (12T-7f) mice were provided by Dr. Robert J. Matusik (Vanderbilt University Medical Center, Nashville, TN [15, 17]). The transgenic construct used large probasin (LPB) promoter to direct the prostatic epithelial cell expression of the SV40 large T antigen (*Tag*) [11], with a deletion in the early region to remove the small t antigen.

Breeders were fed standard pellet mouse feed and water *ad libitum*. After weaning of the animals at 3 to 4 weeks of age, the gender of the offspring was determined, males were separated, and a tail biopsy was collected from each mouse.

Genotypes of offsprings were determined by polymerase chain reaction of DNA isolated from tail biopsies. The sequences of specific primers for the transgene and PCR protocols were provided by the animal model providers. Both genetically modified mice models were backcrossed to C57BL/6J mice for at least 10 generations to ensure a similar genetic background in the mice.

Cross-breeding of LPB-Tag with CTRKO mice yielded four groups of mice: wild type WT (lacked both transgenes), CTRKO-LPB-Tag (both genotypes), CTRKO alone, LPB-Tag alone. Each group had six-ten males. This number was determined by power calculations for *p* < 0.05 as previously described [50, 51]. The sample size was determined to test the hypothesis that CTR-deficiency will interfere with T-antigen-induced prostate tumor formation. The mice were maintained till they reached three months of age (weighed 22–39 g). The mice were weekly monitored for body weight, food and water intake as well as behavioral changes, and sacrificed at the age of three months. At necropsy, their prostate glands were collected on ice, weighed and fixed in neutralized formalin, and processed for paraffin embedding as previously described [19]. The fixed tissue was used either for RNA extraction or for cutting 5 µm thick paraffin sections.

All animal studies conducted in accordance with the established guidelines and protocols for establishing colony as well as for the experimental study were approved by the Institutional Animal Use and Care Committee at the University of Louisiana at Monroe. The protocol conformed to the ‘Guide for Care and Use of Laboratory Animals’ published by the US National Institutes of Health (NIH publication No. 85-23, revised 1996).

### Tissue preparation/histopathologic analysis

#### Histology

Hematoxylin and Eosin staining and immune fluorescence analyses were performed on 5-μm thick prostate sections mounted on Super-frost/Plus slides.

#### Validation of antibodies for immunofluorescence

Antibodies against human CTR have been previously validated for the IHC of prostate cancer specimens [7]. Tissue sections (5 μm) were deparaffinized, hydrated, and CT/CTR immunofluorescence was performed as previously described [14]. Antibodies against T Antigen, Ki 67, fibronectin, vimentin, E- cadherin, ZO-1, vimentin, and Snail were tested for specificity and used as previously described [14, 52].

#### Controls

Tissue sections were incubated either in the presence of no primary antibody, no secondary antibody, or primary antibody blocked with the antigen.

#### Immunofluorescence for CTR and T-antigen

Paraffin sections of tissue blocks were deparaffinized as described earlier and boiled for 10 min in citrate buffer (pH 7.0) for antigen retrieval. The sections were then blocked with normal goat serum and incubated overnight at 4°C with the primary antibody in PBS. Slides were then reacted with antirabbit IgG conjugated with TRITC or FITC. Sections were counterstained with DAPI, dehydrated, mounted, and visualized under a Nikon Optiphot Fluorescent Microscope. Images were captured by Retiga 2100 monochrome camera connected to Mac computer. The microscopic images were processed by Biovision image analysis computer program. The sources and concentrations of primary antibodies used are: anti-CTR (1:250, Acris-Origene, Rockville, MD); T-Antigen (1:200, Cell Signaling, Danvers, MA).

### Proliferation of prostate epithelial cells

To evaluate prostate epithelial cell proliferation, 5-μm-thick, paraffin-embedded tissue sections of the prostate were immunostained for Ki67 using a commercially available rabbit anti-mouse monoclonal antibody (Novus Biologicals, Centennial, CO). This primary antibody, anti-Ki67 was applied at a dilution of 1:500. The secondary antibody was FITC-conjugated anti-rabbit IgG. The number of Ki67- positive cells in 6 randomly selected 400× (i. e., high-power) fields was counted and divided by the total number of cells (Ki67-positive and DAPI-positive) in those fields (*n* = 5/group) to yield the percentage of Ki67-immunopositive cells.

### RNA preparation

Paraffin-embedded mouse prostate blocks were used for RNA extraction. The tissue blocks were deparaffinized and the RNA was extracted using RNAEasy FFPE kit (Qiagen, Germantown, MD). The manufacturer’s instructions were followed. Total RNA was checked for purity and concentration by spectrophotometer measurement (ND-1000, NanoDrop Technologies, Rocky River, OH, USA), and RNA integrity by electrophoresis on Agilent Bioanalyzer 2100 (Santa Clara, CA). Samples were either used for global expression analysis or quantitative real-time PCR analysis. RNA samples used in real-time PCR were reverse transcribed according to manufacturer’s protocol (Ambion, Austin, TX, USA).

### Transcription microarray

One hundred nanograms of total pooled RNA from each of four experimental groups of transgenic mice were used to prepare biotinylated fragmented cRNA with GeneChip™ WT pico Reagent kit according to the instructions provided by the manufacturers (ThermoFisher, Waltham, MA). The biotinylated cRNAs were then hybridized to Clariom D mouse transcriptome Arrays (Affymetrix, Santa Clara, CA). All array experiments were performed by the University of Kansas Medical Center Genomics Core Facility (Kansas City, KS).

### Microarray data analysis

The gene expression data were obtained using the TAC suite, version 4 (ThermoFisher). The expression data were normalized to the target value of 150 by global scaling. This procedure uses a constant scaling factor for every gene on an array, where the scaling factor is obtained from a trimmed average signal of the array after excluding the 2% of the probe sets with the highest and the lowest values. After normalization, the expression profiles were imported into a Microsoft Excel database.

### Quantitative real-time PCR

The cDNA synthesis from total RNA was performed using the Superscript™ II Reverse Transcriptase cDNA synthesis protocol (Invitrogen, CA, USA), followed by SYBR Green quantitative real-time PCR. Each sample was run in triplicates. The quantity of mRNA was calculated based on the threshold cycle (*C*_T_) values which were standardized by the amount of the housekeeping gene. The triplicate average mouse GAPDH *C*_T_ value was subtracted from the corresponding target gene’s *C*_T_ -value, representing the ∆ *C*_T_. Further calculation was performed using the 2^−∆∆CT^ method, always pairwise comparing the target gene with the gene of interest tested. Melt curve and agarose gel electrophoresis analyses were performed to confirm a single product of expected length was amplified. The following primers were used:

GAPDH (Fwd 5′AGGTCCGTTTGAACCGATCT3′; Rev 5′TCGCAGTGAGTGGTGTCATA3′), HMGCS1 (Fwd 5′CCGCAGTTTGGGTGAAGAAA3′; Rev 5′GACAGAATTGGCCAAGCACA3′), HMGCR (Fwd 5′TGCCGTCAATTTCCCAGTTG3′; Rev 5′ATCACATTCGCACGGCTAAC3′),

SCD1 (Fwd 5′TGCACACCACAAATTCTCCG3′; Rev 5′CCAGCAATACCAAGGCACAA3′), DPT (Fwd 5′ACAACAGCCTGGGGTCAATA3′; Rev 5′TGCGTAGTTCCACTGTCTGT3′), AGPS (Fwd 5′GTGATCGGCGAATCGTTTGA3′; Rev 5′CCGTGTGTTCAAAGACCGTT3′), FABP4 (Fwd 5′ATTACCATGTTCACAGGCCC3′; Rev 5′TGGTAGTTCAAGGTCGTGCA3′), and PTEN (Fwd 5′-AATTCCCAGTCAGAGGCGCTATGT-3′; Rev 5′-GATTGCAAGTTCCGCCACTGAA-3′).

### Expression of epithelial and mesenchymal markers

Since CTR is shown to induce EMT in prostate cancer cells, we examined the expression of epithelial markers E-cadherin and ZO-1 as well as mesenchymal markers vimentin and fibronectin in mouse prostate sections. The sources and concentrations of the antibodies were as follows: anti-Vimentin (1:200), anti-E-cadherin (1:200) from Cell Signaling; anti-fibronectin (1:200) from Santa Cruz; anti-zonula occludens 1 (1:200) from Abcam (Cambridge, UK). Mouse prostate sections were also tested for the presence of EMT markers such as snail and N-cadherin. The source and concentrations were: anti-snail (1:100) from Novus Biologicals and anti-N-cadherin (1:400), from Abcam.

### Image analysis and interpretation

Six images per section were acquired at 40× magnification. The prostate tissue antigen expression was scored based on the frequency as well as the intensity of staining (absence of staining, <1+, 1+, 2+, and 3+). Immunostaining was scored by two individuals independently using established methods and the mean reading was taken. An IHC index for each sample was calculated by multiplying staining intensity with the percentage of positive cells. The results were graded from 0 (negative) to 300 (all cells display strong staining intensity). Reproducibility of the analysis was verified by rescoring of randomly chosen slides. Duplicate readings gave similar results. The samples were broadly classified as: i) negative (IHC index = 0); ii) low (IHC index <5); iii) moderate (IHC index = 5-50); iv) high (IHC index = 50-150); and v) very high (IHC index >150).

### Statistical analysis

Statistical calculations were performed using GraphPad Prism™ version 7 software (San Diego, CA). Results are generally expressed as mean IHC index ± standard error of the mean (SEM) unless otherwise stated. *P* < 0.05 was considered to indicate a statistically significant result. One-way ANOVA and *t*-tests were used to compare the appropriate IHC index across the animal groups. Differences between WT and TG groups were calculated using Student’s independent *t* -test and presented as mean ± SEM. A *P* -value of < 0.05 was considered statistically significant.

## SUPPLEMENTARY MATERIALS


