# Correction: Calcitonin receptor is required for T-antigen-induced prostate carcinogenesis

**DOI:** 10.18632/oncotarget.28394

**Published:** 2023-04-14

**Authors:** Ajay Kale, Afaf Aldahish, Girish Shah

**Affiliations:** ^1^Pharmacology, University of Louisiana College of Pharmacy, Monroe, LA 71201, USA

**This article has been corrected:** In Figure 9C, the first panel image (N-Cadherin staining in wild type mice) is an accidental duplicate of the third panel image in Figure 1D. The corrected Figure 9C, produced from the original data, is shown below. The authors declare that these corrections do not change the results or conclusions of this paper.

Original article: Oncotarget. 2020; 11:858–874. 858-874. https://doi.org/10.18632/oncotarget.27495

**Figure 9:**
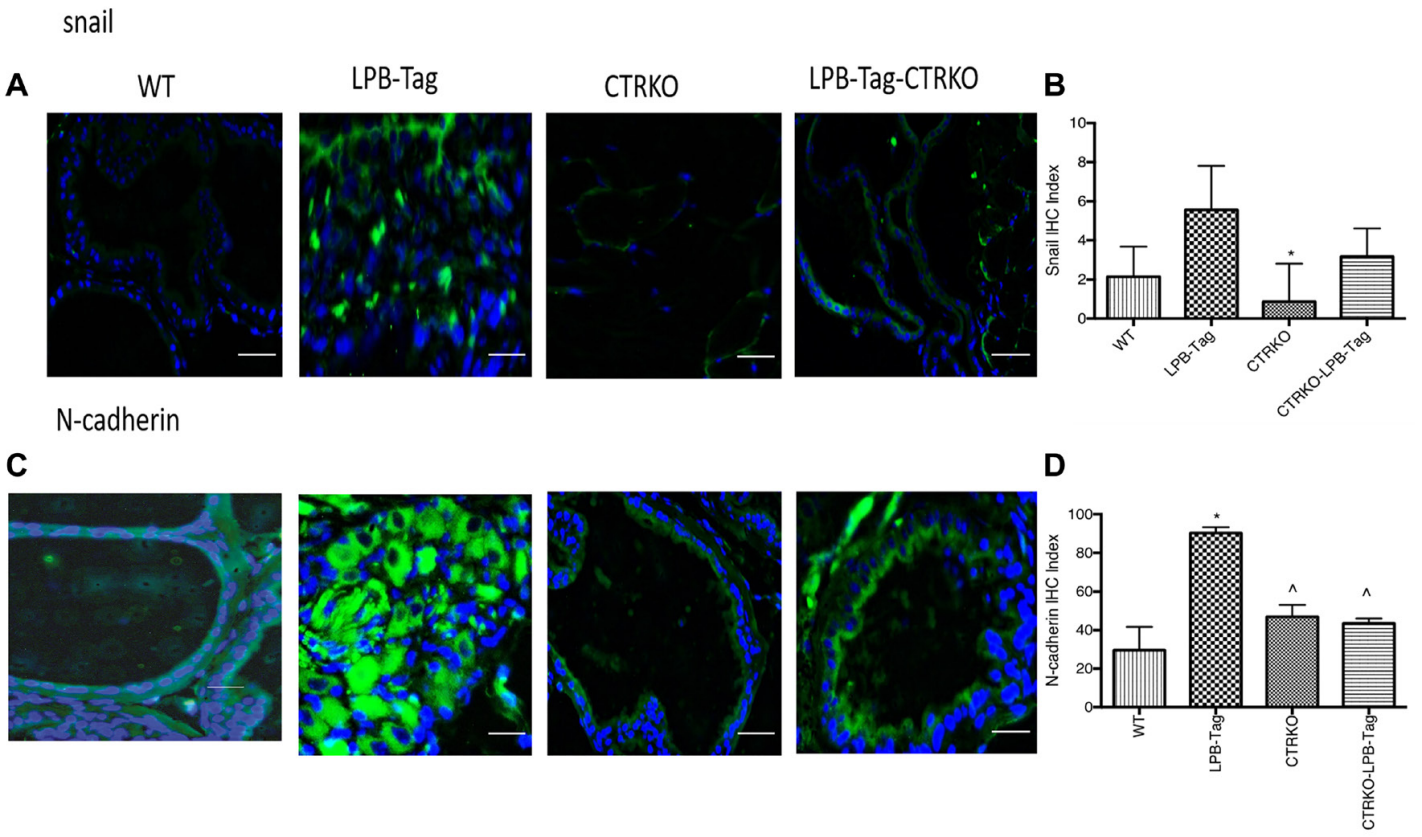
Immunoreactivity of snail and N-cadherin. (**A**) Representative photomicrographs of immunofluorescence for snail in the prostate tissues of WT, CTRKO, LPB-Tag and LPB-Tag-CTRKO mice. Green staining represents snail activity while blue staining represents the DAPI at 40× magnification; Scale bar 100 μm. (**B**) Figure represents mean IHC staining index for snail immunofluorescence observed in the prostate tissues of WT, CTRKO, LPB-Tag and LPB-Tag-CTRKO mice; ^*^represents significantly different than LPB-Tag; *p* < 0.05. (**C**) Representative photomicrographs of immunofluorescence for N-cadherin in the prostate tissues of WT, CTRKO, LPB-Tag and LPB-Tag-CTRKO mice. Green staining represents N-cadherin activity while blue staining represents the DAPI at 40× magnification; Scale bar 100 μm. (**D**) Figure represents mean IHC staining index for the N-cadherin immunofluorescence observed in the prostate tissues of WT, CTRKO, LPB-Tag and LPB-Tag-CTRKO mice; ^*^represents significantly different than WT and ^^^represents significantly different than LPB-Tag; *p* < 0.05.

