# Supplementary material for: Calcitonin receptor is required for T-antigen-induced prostate carcinogenesis
Source: Oncotarget. 2020 Mar 3;11(9):858–74. doi: 10.18632/oncotarget.27495 (PMC7061735; doi:10.18632/oncotarget.27495)
Supplement: Supplementary file 1 [file oncotarget-11-858-s001.pdf]

Calcitonin receptor is required for T-antigen-induced prostate carcinogenesis

SUPPLEMENTARY MATERIALS

Supplementary Table 1: Gene ontology

GO biological process term	Term Hits	All genes	Annotated genes for term	All annotated genes	*P* (Fisher's Exact Test)
Metabolic process	39	67	7166	22296	5.35E-05
Organic substance metabolic process	37	67	6644	22296	6.37E-05
Tissue development	17	67	1625	22296	8.98E-06
Small molecule metabolic process	16	67	1428	22296	7.48E-06
Lipid metabolic process	22	67	1048	22296	6.98E-13
Cellular lipid metabolic process	19	67	799	22296	4.47E-12
Organophosphate metabolic process	10	67	698	22296	6.77E-05
Monocarboxylic acid metabolic process	9	67	456	22296	1.42E-05
Small molecule biosynthetic process	9	67	445	22296	1.17E-05
Supramolecular fiber organization	8	67	444	22296	8.25E-05
Lipid biosynthetic process	13	67	417	22296	7.19E-10
Striated muscle tissue development	7	67	322	22296	7.58E-05
Fatty acid metabolic process	9	67	304	22296	5.65E-07
Epidermis development	8	67	265	22296	2.18E-06
Skin development	8	67	246	22296	1.27E-06
Steroid metabolic process	7	67	220	22296	7.04E-06
Monocarboxylic acid biosynthetic process	5	67	140	22296	9.22E-05
Fatty acid derivative metabolic process	5	67	125	22296	5.49E-05
Actomyosin structure organization	5	67	103	22296	2.26E-05
Fatty acid biosynthetic process	5	67	98	22296	1.79E-05
Cellular component assembly involved in morphogenesis	5	67	97	22296	1.71E-05
Isoprenoid metabolic process	6	67	74	22296	1.89E-07
Muscle fiber development	4	67	66	22296	6.90E-05
Triglyceride metabolic process	4	67	65	22296	6.52E-05
Myofibril assembly	5	67	61	22296	2.00E-06
Sarcomere organization	4	67	41	22296	1.19E-05
Isoprenoid biosynthetic process	5	67	29	22296	6.73E-08
Cardiac myofibril assembly	3	67	25	22296	9.09E-05
Skeletal myofibril assembly	3	67	14	22296	1.93E-05
Skeletal muscle thin filament assembly	3	67	13	22296	1.60E-05
